# Serial Backward Locomotor Treadmill Training Improves Bidirectional Walking Performance in Chronic Stroke

**DOI:** 10.3389/fneur.2022.800757

**Published:** 2022-03-14

**Authors:** Oluwole O. Awosika, Dorothy Chan, Bridget A. Rizik, Heidi J. Sucharew, Pierce Boyne, Amit Bhattacharya, Kari Dunning, Brett M. Kissela

**Affiliations:** ^1^Department of Neurology and Rehabilitation Medicine, University of Cincinnati, Cincinnati, OH, United States; ^2^Division of Biostatistics and Epidemiology, Cincinnati Children's Hospital Medical Center, Cincinnati, OH, United States; ^3^Department of Rehabilitation, Exercise and Nutrition Sciences, University of Cincinnati, Cincinnati, OH, United States; ^4^Biomechanics-Ergonomics Research Laboratories, Department of Environmental Health, University of Cincinnati Medical College, Cincinnati, OH, United States

**Keywords:** backward locomotion, post stroke walking rehabilitation, gait rehabilitation, backward treadmill training, walking impairment

## Abstract

**Background and Research Question:**

Walking impairment remains a major limitation to functional independence after stroke. Yet, comprehensive and effective strategies to improve walking function after stroke are presently limited. Backward Locomotor Treadmill Training (BLTT) is a promising training approach for improving walking function; however, little is known about its mechanism of effect or the relationship between backward walking training and resulting overground forward walking performance. This study aims to determine the effects of serial BLTT on spatial aspects of backward and forward walking in chronic post-stroke individuals with residual walking impairment.

**Methods:**

Thirty-nine adults (>6 months post-stroke) underwent 6 days of BLTT (3 × /week) over 2 weeks. Outcome measures included PRE-POST changes in backward and forward walking speeds, paretic and non-paretic step lengths, and single-support center of pressure distances. To determine the association between BLTT and overground walking, correlation analyses comparing training-related changes in these variables were performed.

**Results:**

We report an overall improvement in BLTT and overground walking speeds, bilateral step lengths, and single-support center of pressure distances over six training sessions. Further, there were weak positive associations between PRE-POST changes in BLTT speed, BLTT paretic step length, and overground forward walking speed.

**Conclusion and Significance:**

Our findings suggest that individuals with chronic post-stroke walking impairment experience improvements in spatial walking measures during BLTT and overground. Therefore, BLTT may be a potential adjunctive training approach for post-stroke walking rehabilitation.

## Introduction

Walking impairment resulting from stroke significantly reduces functional ambulatory independence and is a significant public health issue worldwide (1). While most post-stroke survivors have some residual walking ability, <8% have adequate walking speed and endurance to allow for normal daily functioning (e.g., work, grocery shopping) (2, 3). This insufficient recovery of walking ability is devastating because it leads to a loss of life roles, social isolation, dependency, sedentary lifestyle, and increased risk of falls, fractures, and secondary medical complications (4). It is recognized that post-stroke walking rehabilitation training should be multimodal (3) and emphasize exercises such as task-specific training (5), paretic limb weight-bearing (i.e., strengthening) (6), aerobic conditioning (7), and balance (3); yet, there are currently limited rehabilitative training strategies available that incorporate all of these rehabilitative components into one exercise. In light of the anticipated increase in the rate of post-stroke survivors with residual walking impairment (8), the development of more comprehensive and time-efficient rehabilitation approaches is needed to facilitate the rate and extent of walking recovery.

Recent studies suggest that backward walking training may be one comprehensive rehabilitative approach to enhance walking recovery (9–11). Although the mechanism of backward walking is not well-understood, past electrophysiologic studies in healthy controls and the elderly have reported that backward walking training activates trunk, hip, and knee muscles to a greater extent than forward training. In addition, backward walking training has been suggested to improve motor control by alleviating the maladaptive flexor-synergy gait pattern associated with central nervous system injury (12–14). Further, functional neuroimaging and electrocorticography studies report greater cerebral activity in the supplementary motor area, pre-central gyrus, and superior parietal lobule during backward compared to forward walking, suggesting that backward walking presents more of a challenge to the nervous system and therefore may provide greater neuronal connectivity, which may contribute to enhancing corticomotor plasticity (15–17). From a functional movement perspective, investigations spanning a wide range of individuals, including the young and neurologically intact, elderly, and neurologically impaired individuals, have reported that backward training can improve forward walking performance by promoting lower extremity strengthening (18), enhancing proprioception (10, 19), agility, and balance (11)—ultimately leading to an improvement in overground forward walking speed (20).

Specific to the post-stroke population, a limited number of pilot studies and small randomized controlled have studied backward walking training in combination with other conventional modalities with promising preliminary results. These include backward treadmill training with the use of body-weight support (BWS) (21, 22), with and without usual physical therapy (23, 24), direct overground training with standard physical therapy (25), observation (26), and mirror assisted therapy (27), and concurrent administration of botulinum toxin (28). Despite the wide variations in approaches to backward walking training, the majority of these studies have reported training-related improvements in walking speed, balance, and other spatiotemporal measures (9). Our group recently developed a training protocol termed backward locomotor treadmill training (BLTT) in light of these past studies. BLTT differs from past backward walking training studies in the post-stroke population because the entirety of the training takes place on an instrumented treadmill and does not utilize BWS. Specifically, it is postulated that the absence of body weight support inherently requires trainees to bear more weight on the paretic leg (29) while concurrently receiving a high dose of practice on a continuously moving platform (i.e., treadmill), likely providing a greater exercise than previous backward walking training approaches. Additionally, the use of an instrumented treadmill in this protocol allows for real-time adjustments and longitudinal monitoring of backward walking training speeds and other metrics (e.g., step length) and helps to highlight how these parameters change over time. For example, it is unknown whether trainees with chronic post-stroke walking impairment will overtime increase their backward walking speed or lengthen vs. shorten their step lengths—a factor that may help to understand better the mechanistic benefits of BLTT in relation to walking performance and neuromotor control (30–35). Similarly, the correlation between training-related changes during backward training and overground walking has not been reported and may provide preliminary insight into which aspects of BLTT most closely predict overground walking performance.

Our group recently performed a pilot safety, feasibility, and preliminary efficacy of BLTT and a non-invasive spinal neuromodulatory protocol (36). That study confirmed that the BLTT protocol is safe and feasible in chronic stroke survivors and found clinically meaningful improvement in walking speed lasting beyond 2 weeks post-training with BLTT that was independent of non-invasive neuromodulation. However, due to the limited scope of that study, that manuscript was unable to incorporate outcomes concerning training-related changes associated with BLTT or the relationship between those changes and overground forward walking performance. Hence, this secondary analysis manuscript utilizes data collected from that study along those from nine additional individuals subsequently enrolled into the protocol to optimize statistical power for achieving the aims of this study. As such, the objectives of this manuscript are to highlight training-related spatial changes over six sessions of BLTT in chronic post-stroke individuals, and to determine its association with overground walking performance. Knowledge gained from study may help provide key preliminary insight into the relationship between backwards treadmill training and forward walking performance, while serving as a means for sample-size determination for larger prospective studies.

## Methods

### Design Overview

#### Setting and Participants

This study was approved by the University of Cincinnati Institutional Review and was performed in the Neurorecovery Lab from September 2017 to October 2019. Thirty-nine chronic stroke survivors with residual walking impairment were recruited from the community and gave written informed consent prior to enrollment, in accordance with the recommendations of the Declaration of Helsinki. Inclusion and exclusion criteria as previously described elsewhere (36) were: *Inclusion:* 18–80 years of age, residual walking impairment secondary to ischemic/hemorrhagic stroke(s), >6 months post-stroke (chronic), ability to provide consent, ambulate at least 10 meters without a walker, and maintain at least a 0.13 m/s speed on the treadmill while walking in a backward direction for a duration of six consecutive minutes. In addition, all participants were asked to abstain from formal physiotherapy and botulinum toxin injections at least 2 weeks prior to study enrollment and through the entirety of training and follow-up (37). *Exclusion*: Unstable cardiovascular status precluding participation in a moderate-high intensity exercise, severe lower extremity spasticity (modified Ashworth >2/4), significant language barrier which may interfere with the ability to follow instructions during training and testing, and untreated depression [>10 on the Patient Health Questionnaire (PHQ9)] (38), see Table 1.

**Table 1:** Baseline characteristics of study participants (*n* = 39).

	***n***	**Percentage**	
Gender (female)	17	44	
Stroke type (ischemic)	31	79	
Left hemispheric	19	49	
Brain stem/cerebellar	6	15	
Single point cane	4	10	
Quad cane	6	15	
Hemi-walker	1	3	
AFO	13	33	
KAFO	2	5	
	**Mean**	**Median**	**Range (min–max)**
Age	56.9	57.3	33.7–72.9
Time post-stroke (years)	4.18	2.40	0.70–18.6
PHQ9 score	4.38	3	0–16
MMSE score	28.1	29	18–30
Height (cm)	174	172	157–195
**Gait Measures**			
**Backward locomotor treadmill training**			
Speed (m/s)	0.29	0.27	0.13–0.69
Step length (cm) -paretic leg	25.9	25.0	10.0–61.75
Step length (cm)-non-paretic leg	24.6	23.8	10.0–64.75
Step length symmetry index (%)	94.4	96.7	82.6–100
**10-meter walk test (fast)**			
Speed (m/s)	0.99	1.06	0.19–2.00
Step length (cm)-paretic leg	60.0	60.0	34.8–89.6
Step length (cm)-non-paretic leg	53.5	53.5	14.3–92.1
Step Length Symmetry Index (%)	88.9	92.1	52.7–99.9
SS COP dist. (cm)- paretic leg	6.81	5.00	1.13–16.1
SS COP dist. (cm) - non-paretic leg	10.9	11.1	8.50–18.6
SS COP dist. symmetry index (%)	69.1	68.0	19.3–99.6

*AFO, Ankle-Foot Orthosis; KAFO, Knee-Ankle-Foot Orthosis; MMSE, Mini-Mental State Examination; PHQ-9, Patient Health Questionnaire 9-item Depression Scale; SS COP Dist., Single Support Center of Pressure Distance. Symmetry indices range from 0 TO 100%, where 0% means complete asymmetry and 100% means perfect symmetry*.

#### Description of Training and Outcomes

##### Backward Locomotor Treadmill Training

**Figure 1:**
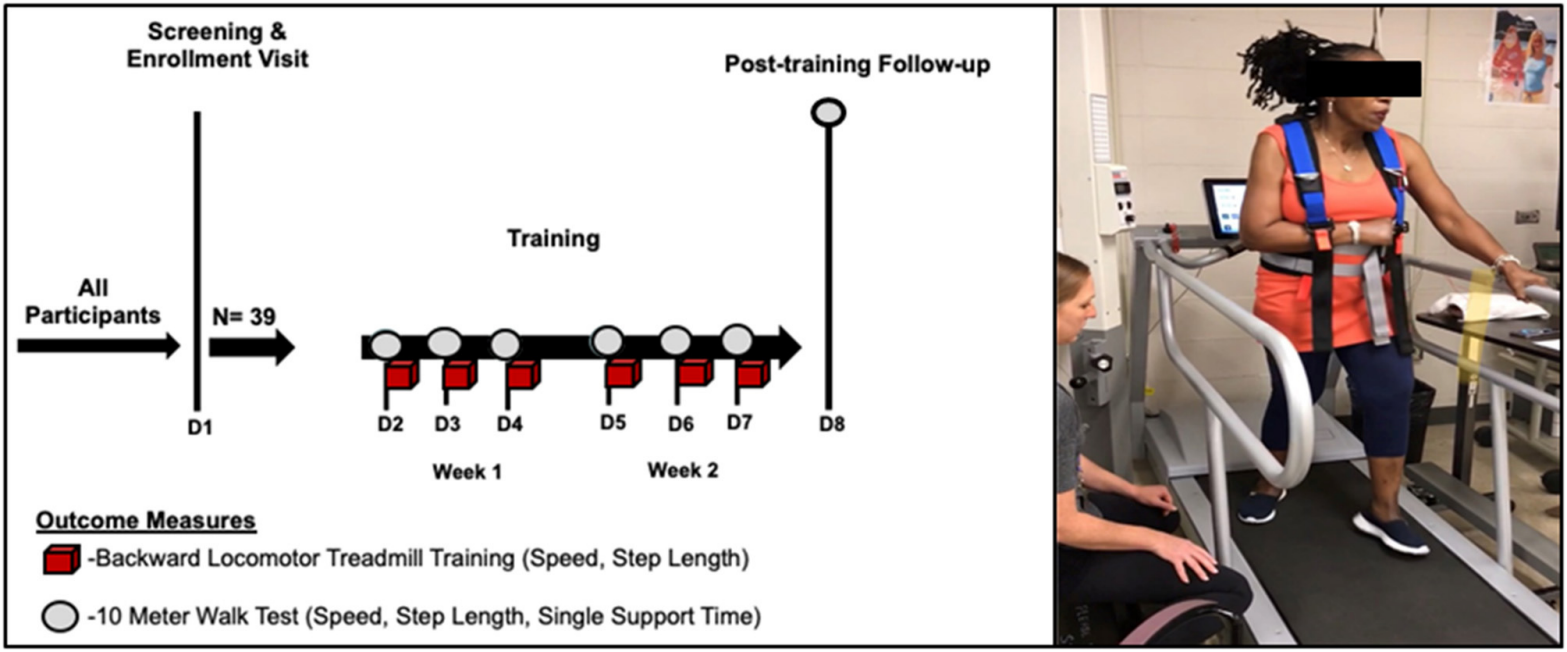
Backward locomotor treadmill training (BLTT) protocol. Study participants underwent six, 30-min sessions of BLTT over a 2-week period (red cubes). Outcome measures were obtained prior to training at baseline (D2), subsequent training days (D3–D7), and ~24 h following the completion of training (D8).

On the first visit (screening), enrolled participants were oriented to the backward locomotor treadmill task while holding one handrail for support for 3 min. All participants were expected to maintain the minimum required training speed of 0.13 m/s on the instrumented Biodex Gait Trainer™ 3 motorized treadmill, per study inclusion criteria, as this speed was the minimum necessary for the treadmill sensors to detect and record walking metrics associated with training. Based on the comfort level, the belt speed was increased in increments of +0.04 m/s until a comfortable training speed was achieved, as previously described (36). Qualifying participants then underwent six sessions of training, which consisted of four 6-min blocks. All training sessions were conducted by a protocol-trained and certified physical therapist, Figure 1. The starting belt speed was based on the last preferred speed achieved on the previous day, with the option to increase or reduce the speed, per subject preference. For additional safety, all participants wore a safety harness (without-body weight support) and were provided 2-min rest breaks between each 6-min training block. Participants were cued to step “reach” back as far as possible with each step during the swing phase of gait while working to maintain an upright posture throughout the duration of the training period. In addition, to reduce possible confounders between training sessions, participants were instructed not to practice walking backward outside of the study protocol for the duration of the study.

#### Outcomes

##### BLTT

The outcome variables obtained during training were the change in backward walking speed and step lengths (paretic and non-paretic). Previous studies have suggested that backward walking ability decreases with age and is characterized by decreasing speeds and stride lengths and may be related to a myriad of factors such as an age-related decline in strength, neuromotor control, and biomechanical constraints (39, 40). Since stroke commonly impacts chronologically older individuals, compounded by acquired functional hemiparesis, it is likely that study participants may experience similar limitations. Hence, investigating training-related changes in these parameters may inform how BLTT may impact lower extremity strengthening and neuromotor control. These measures were acquired using built-in treadmill sensors (41) and were later exported for offline analysis. Four separate values (from each 6-min training block) were averaged to formulate a single cumulative value per training session. Of specific interest were: PRE to POST changes [Day 7 minus Day 2 (baseline)] in average walking speed and step length during BLTT.

##### Overground Walking

Three measures were obtained during the 10-meter walk test (10-mWT): speed, step length (paretic and non-paretic), and single support center of pressure distance [SS COP Dist. (paretic and non-paretic)]. Walking speed has been shown to be a valid, reliable, and good predictor of functional ambulation and community independence (42) and is commonly impaired after stroke. Likewise, bilateral step length is often shorter than in healthy controls and is associated with decreased walking speed and an increased risk of falls (43, 44). The SS COP Dist. measures how body weight is progressed over the foot during single support and has been suggested to be a predictor of hemiparetic gait velocity (45, 46). As such, measurement of training-related changes in SS COP Dist. may provide information about the neuromuscular response involved in maintaining upright balance and forward progression during walking. To obtain the above measures, the 10-mWT was performed daily prior to the start of BLTT and was captured with a 20-feet Zeno Walkway gait analysis mat (Protokinetics, PA, USA) and Protokinetics Movement Analysis Software (PKMAS) (24), and were later exported for offline analysis. Of specific interest were: PRE to POST [Post-training Day 8 (~24 h post Day 7) – Day 2 (pre-training baseline)] changes in walking speed, average step length, and SS COP Dist.

##### Relationship Between BLTT and Overground Walking Performance

To determine the association between BLTT and forward walking performance, correlation analyses were performed comparing PRE to POST changes between 1. ΔBLTT speed vs. Δ10-mWT speed, 2. ΔBLTT step lengths vs. Δ10-mWT speed, 3. Δ-BLTT step lengths vs. Δ-overground walking step lengths, 4. ΔBLTT speed vs. Δ10-mWT speed SS COP Dist and 5. Δ-BLTT step lengths vs. Δ-overground SS COP Dist.

##### Spatial Symmetry

Symmetry indices were calculated for Step Lengths (BLTT and 10-mWT) and SS COP Dist. (10-mWT), PRE to POST using the following equation: [1 - | Paretic – Non-paretic | / (Paretic + Non-paretic)] ^*^ 100%; with possible values ranging from 0–100%, where 0% means complete asymmetry and 100% means perfect symmetry.

### Statistical Analysis

To address the objectives of this study, only participants that completed at least half of the six training sessions were included for BLTT analysis; therefore, one participant from the original safety and feasibility study who did not complete the first day of training was excluded. For forward walking and correlation analysis, one participant whose forward walking data was uninterpretable during forward gait analysis (due to walk-running). Two participants who did not complete the entire 6 days of the study were excluded from the correlation analysis looking at PRE to POST changes. Shapiro-Wilk tests were used to assess for deviations from normal distribution among the continuous variables, and the significance level was set at *p* = 0.05 for all measures. Paired *t*-tests were used to test PRE to POST changes in spatiotemporal measures on the treadmill (backward) and overground (forward). Robust regression was used to limit the impact of outliers in determining the relationship between training-associated changes during BLTT compared to changes observed with the 10-mWT (47).

## Results

### BLTT: Speed

**Figure 2:**
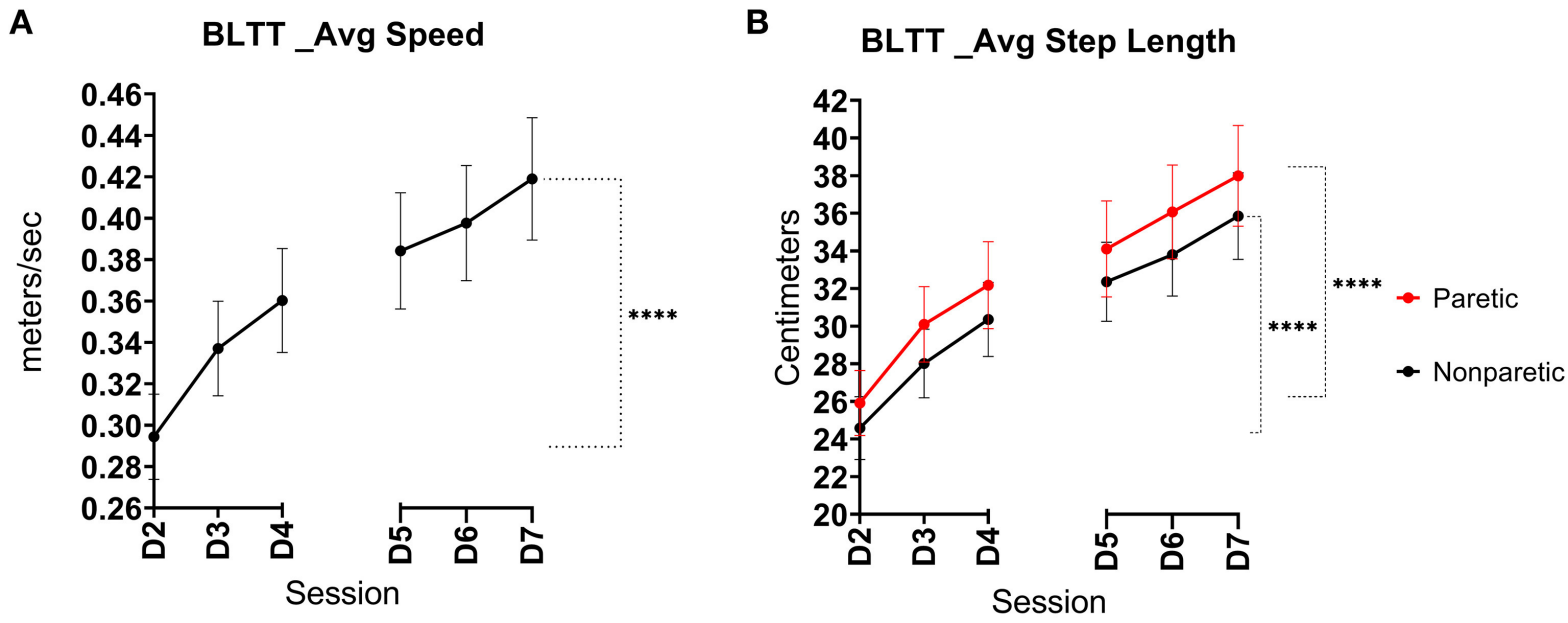
Backward locomotor training (BLTT) speed and step length over six training sessions. Mean progression in BLTT speed **(A)** paretic and non-paretic step lengths **(B)** per training session from baseline (D2) through session 6 (D7), (error bars show standard error, ****indicates significance level <0.001).

The BLTT speed was significantly greater on Day 7 (0.42 m/s ± 0.18) relative to baseline (D2) (0.30 m/s ± 0.13), (*p* < 0.001), see Figure 2A.

### BLTT: Step Length

Both lower extremities demonstrated an improvement in step length. The non-paretic leg showed an increase in step length at Day 7 (35.9 ± 14.0 cm) compared to baseline (24.8 ± 10.7 cm), *p* < 0.001. Similarly, the change in paretic step length on Day 7 also increased (38.0 ± 16.3 cm) relative to baseline (26.1 ± 11.0 cm), *p* < 0.001, See Figure 2B. Interlimb BLTT step length symmetry was less on Day 7 (91.8% ± 5.92) compared to baseline (94.4% ± 4.98), *p* = 0.019.

### 10-mWT: Overground Walking Speed

**Figure 3:**
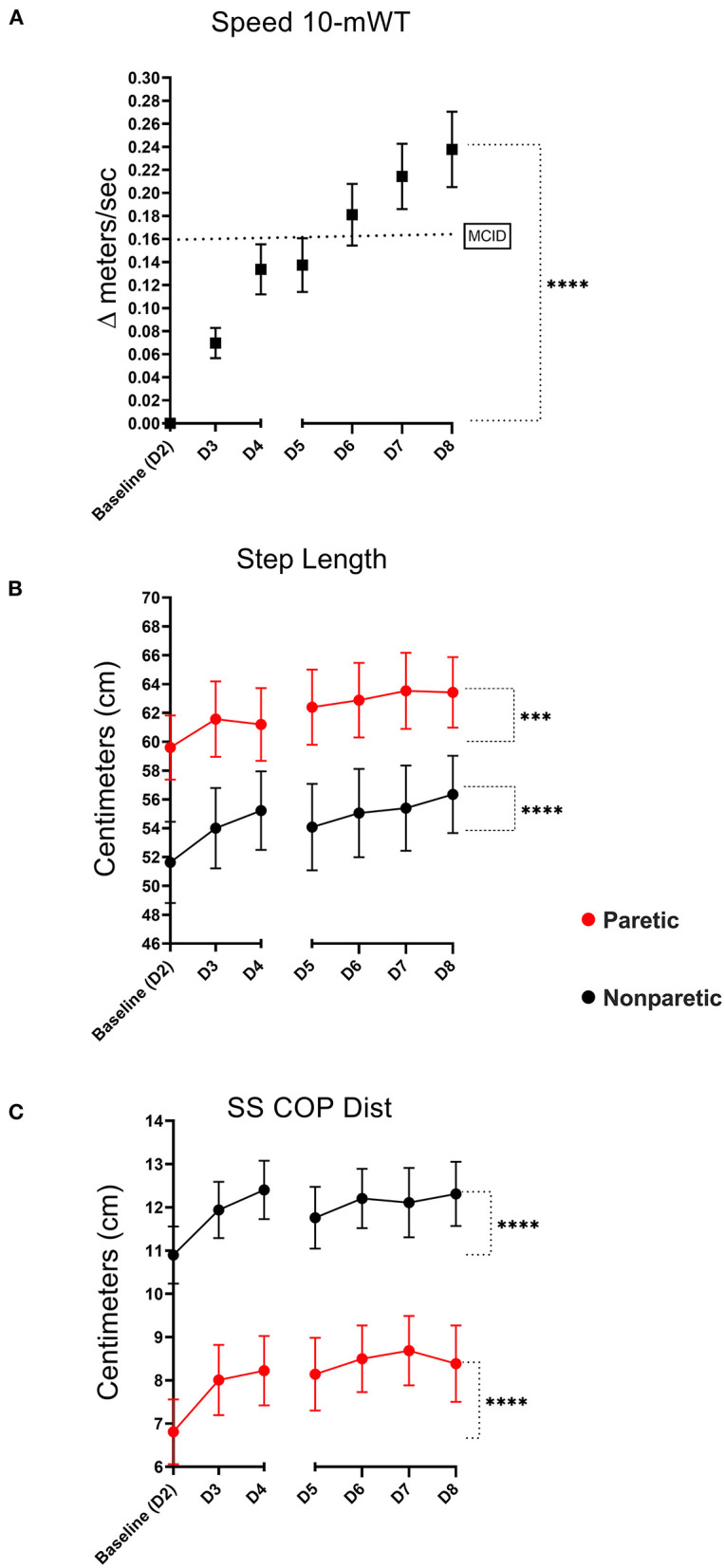
Ten meter walk test (10-mWT). Mean change in 10-mWT speed relative to baseline [dotted line signifies the minimal clinically important difference (MCID)- ≥0.15 meters/s] **(A)**. Mean training-related change in paretic and non-paretic step lengths **(B)**, and percent single support times **(C)** (error bars are standard error, ****indicates significance level <0.001, ***level <0.01).

Overground walking speed was significantly greater on *Post-training Follow-up Day 8* (1.21 m/s ± 0.60) compared to baseline walking speed (0.98 m/s ±0.49), *p* < 0.001, see Figure 3A. In total, 25 of 36 study participants (69%) experienced clinically meaningful improvements in walking speed (≥0.15 m/s) by the completion of training (48).

### 10-mWT: Step Length

Both lower extremities demonstrated an improvement in overground walking step length during overground walking. The non-paretic step length increased from 52.0 ± 17.7 cm at baseline to 56.4 ± 16.1 cm, *P* < 0.001. Likewise, the paretic step length increased from 60.3 ± 13.8 cm to 63.4 ± 14.7 cm (*p* = 0.003), see Figure 3B. Interlimb step length symmetry was greater on Day 8 (90.6% ± 9.48) compared to baseline (88.9% ± 10.8), *p* = 0.019.

### 10-mWT: SS COP Dist

The non-paretic SS COP Dist. increased from 10.9 ± 3.96 to 12.3 ± 4.45 at Post-training Follow-up Day 8, *p* < 0.001. Similarly, the paretic leg also demonstrated an increase from 6.81 ± 4.49 at baseline to 8.38 ± 5.32, *p* < 0.001, see Figure 3C. Interlimb SS COP Dist. symmetry was unchanged on Day 8 (72.8% ± 20.4) relative to baseline (69.1% ± 21.6), *p* = 0.169.

### Correlation Analysis

**Figure 4:**
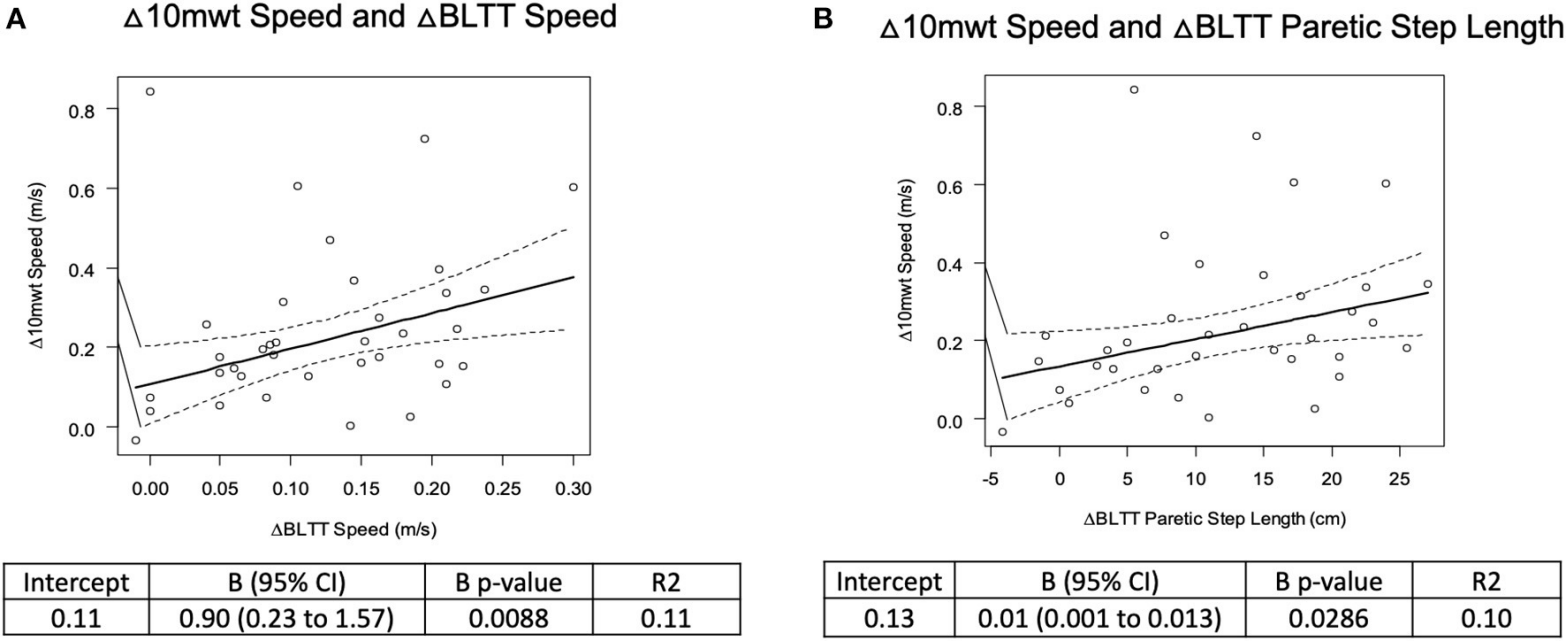
Correlation analysis of backward locomotor treadmill training change in BLTT speed **(A)** and paretic Step Length **(B)** and overground walking speed on the 10-meter walk test.

There was a weak positive relationship between Δ BLTT Speed and Δ 10-mWT Speed, R2 = 0.11, β = 0.90 (0.23–1.57), *p* < 0.010, and ΔBLTT Paretic Step Length and Δ 10-mWT Speed, R2 = 0.10, β = 0.01 (0.001–0.01), *p* < 0.030, see Figure 4. No significant relationship was seen between all the other variables, see Table 2.

**Table 2:** Correlation analysis of backward locomotor treadmill training and overground walking performance.

	**Robust Regression**			
	**Intercept**	**ß (95% CI)**	**ß *p*-value**	**R2**
**Δ10-mwt speed**				
ΔBLTT speed	0.11	0.90 (0.23–1.57)	0.01*	0.11
ΔBLTT non-paretic step length	0.14	0.01 (−0.002 to 0.01)	0.06	0.08
ΔBLTT paretic step length	0.13	0.01 (0.001–0.01)	0.03*	0.10
**Δ10-mwt non-paretic step length**				
ΔBLTT non-paretic step length	5.90	−0.22 (−0.48 to 0.05)	0.11	0.06
**Δ10-mwt paretic step length**				
ΔBLTT paretic step length	3.13	0.10 (−0.12 to 0.31)	0.38	0.02
**Δ10-mwt %SST paretic**				
ΔBLTT non-paretic step length	0.95	0.10 (−0.04 to 0.24)	0.18	0.03
**Δ10-mwt %SST non-paretic**				
ΔBLTT paretic step length	0.51	0.12 (−0.01 to 0.25)	0.08	0.06

## Discussion

Our findings suggest that chronic stroke individuals with residual walking impairment experience progressive improvement in backward walking speed and bilateral step lengths with training. In addition, study participants demonstrated training-related improvement in overground forward walking speed, bilateral step lengths, and single support center of pressure distances. Furthermore, correlation analysis suggests a weakly positive association between the changes in backward walking speed, BLTT paretic step length, and change in overground forward walking speed. In addition, there was a slight improvement in overground step length symmetry following six training sessions.

Previous studies have reported that backward walking ability significantly declines with age, and hemiparesis as a result of stroke increases this likelihood, resulting in functional walking impairment and increased risk of falls (49). Therefore, the finding that our study participants showed improvement in backward walking ability with serial training is encouraging and may have several functional, safety, and quality of life implications (39, 50). Further, while the relationship between backward and forward walking is not entirely understood, previous work suggests that the two forms of locomotion may overlap, enabling one training modality to improve the other (13, 51). As such, in this study, participants demonstrated improvements in both backward and forward walking speeds and step lengths over consecutive days of training.

The mechanism of walking impairment after stroke is multifactorial and it is widely recognized that post-stroke walking rehabilitation training should be multimodal in its approach (3). While our reported findings are preliminary, we postulate that BLTT may be one such approach, as it provides task-specific training (5), facilitates aerobic conditioning (7, 52) and has been suggested to improve balance by emphasizing sensorineural integration practice (3, 25, 50, 53, 54). Moreover, BLTT likely improves walking performance by training key muscles essential for efficient biomechanics during forward walking. For example, previous backward walking exercise studies have demonstrated that the backward walking approach uniquely activates fundamental supplementary core and lower extremity muscle groups, including hip extensors, which are also important contributors to forward walking (25, 55). In addition, since BLTT requires trainees to bear a significant portion of their body weight during training, serial BLTT may improve lower extremity loading ability, resulting in greater stability, postural control, and adequate weight shifting during stance. The observed improvement in SS COP Dist. during forward walking supports the idea that training enables greater lower extremity strengthening and foot and ankle stability, enabling body weight to progress over the foot more effectively during single support (46). Another unifying association is the possibility that BLTT improves neuromotor control, as suggested by the incremental increase in overground step length and symmetry following six training sessions (56)–an attribute that has implications for the stability of gait and is decreased with aging and after brain injury (39, 57).

While improvement in backward treadmill training ability does not inherently translate to improved overground walking performance, our correlation analysis found a positive but weak association between changes in backward treadmill training speed, paretic step length, and forward walking speed. This finding suggests that several confounding variables still exist in determining the precise relationship between backward training and overground forward walking. Nevertheless, these results lay a foundation for future studies that will confirm these relationships and ultimately uncover the best predictors of walking rehabilitation training to improve walking performance.

### Limitations and Future Directions

Our findings are limited by the lack of a control group (forward walking training); therefore, a determination regarding the uniqueness of our measures to BLTT cannot be made. Nevertheless, it is reassuring that previous backward walking protocols with forward walking controls report similar improvements (20). Furthermore, the generalizability of our results is limited due to the single site and exploratory nature of this study; hence future hypothesis-driven, and larger randomized controlled multisite studies are needed to validate our findings. In addition, since the factors contributing to stroke walking impairment are often heterogeneous (i.e., age, stroke size type and location, level of spasticity), larger studies would enable further subgroup analysis to determine the impact of such variables on BLTT performance and overground walking performance. In addition, this study was limited to ambulators; therefore, our findings are not generalizable to non-ambulatory stroke survivors, who BLTT is likely not feasible without the use of bodyweight support. With respect to underlying mechanisms of improvement, the instrumented treadmill used in this study was not equipped with built-in force-sensors, therefore it was not possible to obtain COP-related measures during BLTT. Therefore, future investigations are needed to capture SS COP Dist. during BLTT and empirically determine its influence on overground walking performance. To this end, our conclusion regarding muscle-strengthening and foot-ankle stability was based on observations from previous studies and indirectly from the observed improvement in SS COP Dist. during overground walking. Therefore, future studies should incorporate electromyography and dynamometry to empirically test changes in muscle recruitment patterns and strength associated with BLTT in the post-stroke population. Lastly, while this study was primarily focused of walking speed as the primary outcome measure, clinical measures such as balance (58) and spasticity (59) play an equally critical role in walking rehabilitation and outcome; therefore future BLTT studies should consider adding these measures in order to provide a more comprehensive view of the impact of this training approach on walking rehabilitation.

## Conclusion

To our knowledge, this study is the first to report progressive BLTT-specific changes in the post-stroke population and highlight its association with changes in overground walking performance. Well-powered, prospective randomized control studies are needed to determine the efficacy of BLTT. Likewise, mechanistic-based studies will help determine more definitively which training metrics to target during backward walking training—an essential step in developing and optimizing future stroke rehabilitation clinical trials (60).

## Data Availability Statement

The raw data supporting the conclusions of this article will be made available by the authors, without undue reservation.

## Ethics Statement

The studies involving human participants were reviewed and approved by University of Cincinnati Institutional Review (IRB). The patients/participants provided their written informed consent to participate in this study. Written informed consent was obtained from the individual(s) for the publication of any potentially identifiable images or data included inv this article.

## Author Contributions

OA, DC, BR, HS, PB, and KD: conception and design of the study, acquisition of data, or analysis and interpretation of data. OA, HS, PB, AB, KD, and BK: drafting the article or revising it critically for important intellectual content. All authors contributed to the article and approved the submitted version.

## Funding

Funding for this study was provided by the University of Cincinnati Gardner Institute and an American Academy of Neurology Career Development Award.

## Conflict of Interest

The authors declare that the research was conducted in the absence of any commercial or financial relationships that could be construed as a potential conflict of interest.

## Publisher's Note

All claims expressed in this article are solely those of the authors and do not necessarily represent those of their affiliated organizations, or those of the publisher, the editors and the reviewers. Any product that may be evaluated in this article, or claim that may be made by its manufacturer, is not guaranteed or endorsed by the publisher.
