# Supplementary material for: Serial Backward Locomotor Treadmill Training Improves Bidirectional Walking Performance in Chronic Stroke
Source: Front Neurol. 2022 Mar 14;13:800757. doi: 10.3389/fneur.2022.800757 (PMC8963981; doi:10.3389/fneur.2022.800757)
Supplement: Supplementary file 1 [file Table_1.docx]

**Supplementary Table 1.** Between Group Comparison of Outcomes

|  | **Anodal tsDCS** | **Sham tsDCS** |  |
| --- | --- | --- | --- |
| **Outcome Measures** | **∆ POST-PRE** | | **P-Value** |
| **Backward Locomotor Treadmill Training** |  |  |  |
|  |  |  |  |
| *Speed (m/s)* | 0.127 | 0.122 | 0.773 |
| *Step Length _Paretic_ (cm)* | 11.35 | 12.09 | 0.698 |
| *Step Length _Nonparetic_ (cm)* | 10.99 | 11.27 | 0.855 |
|  |  |  |  |
|  |  |  |  |
| **10-Meter Walk Test** |  |  |  |
|  |  |  |  |
| *Speed (m/s)* | 0.218 | 0.253 | 0.382 |
| *Step Length _Paretic_ (cm)* | 3.658 | 2.870 | 0.684 |
| *Step Length _Nonparetic_ (cm)* | 3.123 | 5.320 | 0.253 |
|  |  |  |  |
| *SS COP Dist _Paretic_ (cm)* | 1.609 | 1.704 | 0.903 |
| *SS COP Dist _Nonparetic_ (cm)* | 0.749 | 1.941 | 0.085 |

*SS COP Dist- Single Support Center of Pressure Distance
